# Blood Pressure Control among Hypertensive Diabetic Patients on Follow-Up at Chronic Clinic of Nekemte Referral Hospital in West Ethiopia

**DOI:** 10.1155/2020/7526257

**Published:** 2020-06-19

**Authors:** Mohammed Gebre Dedefo, Dureti Bayisa Gemechu, Ginenus Fekadu, Tesfa Tekle Dibessa

**Affiliations:** ^1^Clinical Pharmacy Unit, Department of Pharmacy, Institute of Health Sciences, Wollega University, Nekemte, Oromia, Ethiopia; ^2^Birroolee Pharmacy, Nekemte, Oromia, Ethiopia; ^3^Pharmacology Unit, Department of Pharmacy, Institute of Health Sciences, Wollega University, Nekemte, Oromia, Ethiopia

## Abstract

**Background:**

Hypertension is a prevalent comorbid condition in diabetes, affecting ∼20–60% of patients with diabetes, depending on obesity, ethnicity, and age. Adults with diabetes historically have two or three times higher rate of cardiovascular disease (CVD) than adults without diabetes.

**Objective:**

The aim of this study was to assess blood pressure (BP) control and its predictors among hypertensive diabetic patients on follow-up at the chronic clinic of Nekemte Referral Hospital (NRH) in West Ethiopia.

**Methods:**

A cross-sectional study was conducted among hypertensive adult patients comorbid with diabetes taking antihypertensive drugs for at least one year in NRH. Both bivariable and multivariable analyses were done. The odds ratio, along with 95% confidence level, was estimated to identify factors associated with uncontrolled BP by using multivariable logistic regression analysis. The level of statistical significance was declared at *p* value <0.05 levels. The patient's written informed consent was obtained after explaining the purpose and procedures of the study.

**Results:**

A total of 186 study participants were included in this study. The mean age of the participants was 51.2 ± 12.2 years. Blood pressure and blood glucose were controlled in 104 (55.9%) and 106 (57.0%) study participants, respectively. In the multivariable analysis, age ≥60 years (AOR = 4.537, 95% CI = 1.142–18.024, *p*=0.032), duration with hypertension ≥5 years (AOR = 3.534, 95% CI = 1.062–11.760, *p*=0.040), cigarette smoking (AOR = 7.697, 95% CI = 2.356–25.146, *p*=0.001), nonadherence (AOR = 6.584, 95% CI = 2.337–18.553, *p* < 0.001), and uncontrolled glycaemia (AOR = 21.630, 95% CI = 8.057–58.070, *p* < 0.001) were independent predictors of uncontrolled blood pressure.

**Conclusion:**

Compared to the previous studies, BP was better controlled among hypertensive diabetic patients in the present study. Older age, longer duration with hypertension, cigarette smoking, nonadherence, and uncontrolled glycaemia were predictors of uncontrolled BP. Thus, interventions on modifiable factors should be done to improve BP control of patients' comorbid with diabetes.

## 1. Introduction

Hypertension is an extremely common comorbid condition in diabetes, affecting ∼20–60% of patients with diabetes, depending on obesity, ethnicity, and age [1]. Good diabetes mellitus management helps people with diabetes mellitus to live long and have high-quality lives in addition to appropriately managing diabetes and cardiovascular disease risk factors such as hypertension and hypercholesterolemia with a healthy diet, recommended levels of physical activity, and correct use of medicines as appropriately prescribed by a physician [2].

Hypertension is often present as part of the metabolic syndrome of insulin. The risk of both macrovascular and microvascular complications increases with the presence of hypertension [3–5]. The rate of cardiovascular disease (CVD) is two or three times higher in adults with diabetes than adults without diabetes [6]. The risk of CVD increases continuously with rising fasting plasma glucose levels, even before reaching levels sufficient for a diabetes diagnosis [7, 8].

In the United States (US), around 30.3 million adults have diabetes (9.4% of the US population) [9]. Hypertension is a common comorbidity in these patients; it is 1.5 to three times more common in patients with diabetes than in those who do not have the disease [5, 10].

The eighth report of the Joint National Committee (JNC 8) recommends a target BP of <140/90 mm·Hg in patients with diabetes and most patients will require two or more antihypertensive medications to achieve goal BP [11–14]. A quality BP management includes expanding patient and healthcare provider awareness, appropriate lifestyle modifications, access to care, evidence-based treatment, a high level of medication adherence, and adequate follow-up. BP management also requires the engagement of patients, families, providers, and healthcare delivery systems [12, 15, 16].

Multiple studies indicate common risk factors for poor control of hypertension include older age, obesity, longer duration with hypertension, nonadherence, smoking, alcohol intake, physical inactivity, chronic kidney disease, black race, and uncontrolled diabetes mellitus. Treatment of hypertension involves improving medication adherence, detection, and correction of secondary hypertension and addressing other patient characteristics [3, 5, 15–23].

Hence, this study aimed to determine the level of BP control and factors associated with uncontrolled BP among hypertensive diabetic patients, which will help the health care providers and regulatory body to work towards improving the management of hypertension in diabetic patients.

## 2. Materials and Methods

### 2.1. Study Design, Period, and Participants

A cross-sectional study was conducted for two months, from July 05 to September 2, 2018. All adult patients of hypertension with diabetes who attended chronic clinics of the NRH were the source of population, and all adult patients of hypertension with diabetes who attended chronic clinics of the NRH during the study period were the study population.

### 2.2. Inclusion and Exclusion Criteria

All adult patients of hypertension with diabetes taking antihypertensive drugs for at least one year in NRH who came for follow-up during the study period and willing to participate were included in the study. Mentally disabled, those who are unable to hear, and seriously ill patients were excluded from this study.

### 2.3. Sample Size Determination and Sampling Procedure

The required sample size was calculated using single population proportion formula, and the following assumptions were used in order to calculate the required sample size, 43.51% population proportion of controlled blood pressure [24], 95% confidence interval, and marginal error of 5% to get a sample size of 378. Since the total number of hypertensive diabetic patients in NRH was 304, the sample size was adjusted by using correction formula. The calculated sample size was nf = 169. Considering a 10% nonresponse rate, 186 hypertensive diabetic patients were included in the study.

### 2.4. Data Collection and Processing

Data were collected using an interviewer-administered pretested questionnaire that was adopted from different literatures [17–27], and a medical chart review was used to determine different variables. The questionnaire was prepared originally in English which had four parts: sociodemographic characteristics, clinical characteristics, knowledge, and adherence parts. The questionnaire was translated to Afan Oromo which is the local language for the purpose of data collection and it was translated back to English again for consistency. All patients suffering from hypertension and diabetes alone that visited the consultant outpatient clinics of the chronic care center of NRH were interviewed.

### 2.5. Data Analysis and Interpretation

Data were entered into Statistical Package for the Social Sciences (SPSS) version 20.0 (IBM/SPSS, Inc., Chicago, IL) for analysis. Both bivariable and multivariable analyses were done. The odds ratio, along with a 95% confidence level, was estimated to identify factors associated with uncontrolled BP by using multiple stepwise backward logistic regression model. The level of statistical significance was declared at *p* value <0.05 levels.

### 2.6. Operational Definitions

#### 2.6.1. Uncontrolled BP

Systolic and diastolic BPs were recorded during patients' visits, in a seated position, after a rest of at least 5 minutes. Two measures were recorded, in both arms, at 5-minute intervals, and the mean was recorded. The BP recordings at each visit were recorded retrospectively from patients' medical records, and then uncontrolled BP was determined by the average BP recordings across the six (6) months. Systolic BP of ≥140 mmHg and/or diastolic BP of ≥90 mmHg were used as uncontrolled BP [11].

#### 2.6.2. Uncontrolled Blood Glucose

Fasting blood glucose was >130 mg/dL [2].

#### 2.6.3. Knowledge about Hypertension

Knowledge of hypertension was assessed by using a validated fact questionnaire about hypertension that was adopted from studies conducted by Pirasath et al. [25]. There were a total of 14 knowledge questions like knowing normal values of BP as 120/80 mmHg, increase in BP > 140/90 mmHg called HTN. HTN can progress along with age. Both sexes have an equal chance of developing HTN. HTN is a treatable condition. There is a risk of developing HTN if there is a family history of HTN. Aging is a greater risk of HTN. Smoking is a risk factor for HTN. Eating fatty foods is a risk factor for HTN. Overweight is a risk factor for HTN. Regular physical exercise reduces HTN. More salt consumption increases BP. Medication is alone in controlling HTN and HTN can lead to life-threatening condition. The score value of all patients was calculated and the mean was taken out of the total participants' score. The mean of the total score was 11.4. The respondents will be considered as having good knowledge if they answer ≥11.4 and poor if they answer <11.4 of knowledge questions.

#### 2.6.4. Adherence

There were a total of 13 medication adherence questions in the data collection tool that was adopted from studies conducted by Obirikorang et al. [26] and Joho [27]; the question contains 13 items measuring treatment compliance and lifestyle compliance. Of the 13 items, medication regimen compliance is composed of 8 items, and lifestyle compliance is composed of 5 items. Medication regimen compliance includes questions like asking how often they forgot to take their medicine, did they stop taking their medicine because they felt better, because they felt worse, because they believed that medicine was ineffective, because they feared side effects, because they tried to avoid addiction, because of religious beliefs or they were using traditional medicine, and because of cost of medication. The responses were measured on a 4-point Likert scale (every day, frequently, rarely, or never). Lifestyle compliance questions included how often they did smoke, consumed alcohol, engaged in physical exercise, ate table salt, and ate meat with high animal fats. Participants were asked to respond to the single question based on a 4-point Likert scale: how often do desirable or undesirable behaviors relate to control of hypertension. The responses were (1) every day, (2) frequently, (3) rarely, or (4) never. Some questions were set such that the highest score did not reflect the worst scenario of noncompliance. To resolve these questions scores were reversed. For example, how often do you engage in physical exercise (4) every day, (3) frequently, (2) rarely or (1) never. The 13 items measuring treatment compliance and life style compliance were added up to get the sum index with a distribution ranging from 28 to 52 with mean 40.8 (SD = 4.24); the median split was used (42.1), which was dichotomized into two groups, i.e., 1 = those who are nontreatment compliant and 2 = treatment compliant which was 28–41 and 42–52, respectively.

### 2.7. Ethical Approval

Ethical clearance was obtained from the Institutional Research Ethics Review Committee of Wollega University, Institute of Health Sciences. This committee wrote a formal letter of permission to Nekemte Referral Hospital to seek its cooperation. Permission was obtained from the medical director's office of the hospital. The patient's written informed consent was obtained after explaining the purpose and procedures of the study. Data on the sociodemographic and clinical characteristics, knowledge, and adherence of the patients were collected to evaluate the blood pressure control and its determinants. Confidentiality was ensured during the data collection. Thus the name of the patient was not recorded.

## 3. Results

A total of 186 study participants were included in this study, from which 83 (44.6%) were males and 103 (55.4%) were females. The mean age of the participants was 51.2 ± 12.2 years. The largest groups by age were those from 40 to 59 years old, which constituted 101 (54.3%) patients. More than half (53.8%) of the respondents were from rural resident (Table 1).

This study identified that out of all antidiabetic therapy, oral hypoglycemic agents alone 103 (55.4%) were the most prescribed agents while 72 (38.7%) patients were on insulin alone and only 11 (5.9%) were taking both. The majority of the study participants had poor knowledge about hypertension 120 (64.5%) and were nonadherent to their treatment (128 (68.8%)). BP and blood glucose were controlled in 104 (55.9%) and 106 (57.0%) study participants, respectively. It was also observed that most of the patients (73.1%) had no comorbidity. The majority of the participants, 120 (64.5%), were on a single antihypertensive agent (Table 1).

In overall observation, the highest used antihypertensive drug by the study participants was ACE inhibitors 102 (54.8%) followed by diuretics 91 (48.9%), calcium channel blockers 47 (25.3%), and *β*-blockers 27 (14.5%).

In bivariable logistic regression analysis, smoking, nonadherence and uncontrolled glycaemia were associated with uncontrolled BP. Variables with *p* < 0.25 (age, marital status, knowledge of hypertension, duration with hypertension, cigarette smoking, presence of comorbidities, adherence, antidiabetic medications, antihypertensive medications, and glycemic control) were entered into the multiple stepwise backward logistic regression model to identify independent predictors of uncontrolled BP. In the multivariable analysis, age ≥60 years (AOR = 4.537, 95% CI = 1.142–18.024, *p*=0.032), duration with hypertension ≥5 years (AOR = 3.534, 95% CI = 1.062–11.760, *p*=0.040), cigarette smoking (AOR = 7.697, 95% CI = 2.356–25.146, *p*=0.001), nonadherence (AOR = 6.584, 95% CI = 2.337–18.553, *p* < 0.001), and uncontrolled glycaemia (AOR = 21.630, 95% CI = 8.057–58.070, *p* < 0.001) had shown statistical significance in predicting uncontrolled BP (Table 2).

## 4. Discussion

The present study found that BP was controlled in 55.9% of hypertensive diabetic patients. The BP control in this finding was comparable with studies from South Africa (57%) [28] and Chile (59.7%) [29]. BP control is better than the previously done studies in the Jimma University Medical Center (43.51%) [24], Addis Ababa (19.4%) [30], South Africa (42%) [31], India (37.66%) [32], Palestine (23.9%) [33], Malaysia (23.5%) [34], and the USA (49%) [35]. This could be due to concomitant use of oral hypoglycemic agents with angiotensin converting enzyme inhibition being associated with greater responses of blood pressure [36], and this was evident from the finding of this study, that the use of ACE inhibitors was seen in 54.8% of the patients. BP control in this study compared to previous studies also suggests improvements in hypertension management.

The most commonly prescribed drugs in the study were ACEI, followed by diuretics in 54.8% and 48.9% of patients, respectively. These findings were similar to studies from Ethiopia, India, Palestine, and Chile [20, 22, 29, 33]. The guidelines that also recommend treatment for hypertension should include drug classes demonstrated to reduce cardiovascular events in patients with diabetes: ACEIs, ARBs, thiazide-like diuretics, or dihydropyridine CCBs. Multiple-drug therapy is generally required to achieve BP targets [11, 37]. However, different from the guideline, the majority of the study participants (64.5%) were on a single antihypertensive agent. This is evidence for the poor practice of adhering to the guideline by the physicians which has to be improved.

Uncontrolled BP was more likely to occur in patients with age ≥60 years. This finding is consistent with studies from Ethiopia [24], South Africa [38], Zimbabwe [39], and Malaysia [34]. Studies indicate that the reason for uncontrolled BP in older age is due to an interaction between biological factors such as autonomic imbalance and vessel stiffening [40] and behavioral factors that suggest older people have decreased physical activity practice [41].

This study showed that patients with a longer duration of hypertension had a significant association with uncontrolled BP. Similar findings were reported from Ethiopia [24] and China [42]. The reason could be due to that a long duration of treatment period might compromise the patient's beliefs about medication effectiveness [43].

In the present study, cigarette smoking was significantly associated with uncontrolled BP. This finding is consistent with studies from Zimbabwe [39], Spain [44], and the USA [45]. Studies reported that cigarette smoking is not only a cause for uncontrolled hypertension, but it is also a risk of developing cardiovascular complications [46, 47]. Smoking induces endothelial dysfunction, vasoconstriction, insulin resistance, and dyslipidemia [47]. Thus, patients with hypertension comorbid with diabetes had to avoid cigarette smoking to achieve an optimal BP.

In this study, nonadherence was significantly associated with uncontrolled BP and nonadherent patients were around 6.584 times more likely to have uncontrolled BP than patients adherent to medications. A similar finding was reported from the study done in Ethiopia [24] and South Africa [28, 38]. The reason for nonadherence could be the cost of the medications; patients might stop taking their medication when their symptoms were under control; a distance of the hospital from their home; unavailability of medications in the health facilities; side effects. Nonadherence is a major cause of uncontrolled hypertension over the world leading to inappropriate drug dose or class changes, which may lead to increased adverse effects and medical costs [48]. Thus, factors related to the patients that are responsible for nonadherence have to be solved by providing education for the patients and factors related to the facility have to be solved by the government by supplying sufficient medications and building a hospital for the community to avoid long distance.

Patients with poor glycemic control were more likely to have uncontrolled BP. Similar findings were reported from Ethiopia [24], Brazil [49], and Iraq [50]. The possible explanation is, in patients with poor glycemic control, the level of insulin could be lowered, and this lowered level of insulin resulted in the loss of insulin's vasodilator action that contributes to the rise in BP [51]. The other explanation could be that the impaired regulation of the renin-angiotensin system, the sympathetic nervous system, and possibly, endothelial factors in patients with poor glycemic control could be a contributing factor for uncontrolled hypertension [51].

### 4.1. Strength and Limitations

This study was the first study conducted at Nekemte Referral Hospital located in West Ethiopia to determine blood pressure control among hypertensive diabetic patients. This study used data from patients' cards and face to face interviews which allowed us to have complete information to determine blood pressure control and identify associated factors.

Limitations of this study were that self-reporting was used for measuring adherence and knowledge about hypertension. This method has the disadvantages of recalling bias and eliciting only socially acceptable responses and hence, may lead to the overestimation of some of the results, the short period, and small sample size. FBG was used to determine blood glucose instead of glycated hemoglobin due to inaccessibility. Since there is only one referral hospital in West Ethiopia during the study period, this study was done only in a single site.

## 5. Conclusion

Compared to the previous studies, BP was better controlled among hypertensive diabetic patients in the present study. Older age, longer duration with hypertension, cigarette smoking, nonadherence, and uncontrolled glycaemia were predictors of uncontrolled BP. Thus, interventions should be done on modifiable factors to improve BP control of patients' comorbid with diabetes. This could be achieved through pharmacists whose responsibility is to deliver continuing medical education in the field of current pharmacotherapy and more strict control of BP is needed to reduce severe complications of diabetes and hypertension.

## Figures and Tables

**Table 1:** Sociodemographic and clinical characteristics of hypertensive diabetic patients on follow-up at NRH, West Ethiopia, 2018 (*n* = 186).

Sociodemographic and clinical characteristic variables		Frequency	Percent
Sex	Male	83	44.6
	Female	103	55.4

Age in years	18–39	33	17.7
	40–59	101	54.3
	≥60	52	28.0

Marital status	Single	21	11.3
	Married	126	67.7
	Divorced	23	12.4
	Widowed	16	8.6

Residence	Urban	86	46.2
	Rural	100	53.8

Educational status	No formal education	82	44.1
	Primary school	44	23.7
	Secondary school	24	12.9
	College/university	36	19.4

Occupation	Employed	143	76.9
	Unemployed	43	23.1

Duration with hypertension	<2 years	57	30.6
	2–4 years	54	29.0
	≥5 years	75	40.3

Knowledge of hypertension	Good	66	35.5
	Poor	120	64.5

Cigarette smoking status	Yes	25	13.4
	No	161	86.6

Alcohol drinking status	Yes	41	22.0
	No	145	78.0

Adherence	Adherent	58	31.2
	Nonadherent	128	68.8

Presence of comorbidities	Yes	50	26.9
	No	136	73.1

Antidiabetic medications	OHAs alone	103	55.4
	Insulin alone	72	38.7
	Both OHAs and insulin	11	5.9

BP control	Controlled	104	55.9
	Uncontrolled	82	44.1

Glycemic control	Controlled	106	57.0
	Uncontrolled	80	43.0

Antihypertensive medications	Single AHAs	120	64.5
	Two AHAs	46	24.7
	Three AHAs	20	10.8

OHAs = oral hypoglycemic agents; AHAs = antihypertensive agents.

**Table 2:** Bivariable and multivariable analysis of factors associated with uncontrolled BP among hypertensive diabetic patients on follow-up at NRH, West Ethiopia, 2018 (*n* = 186).

Variables		Blood pressure		COR (95% CI) *p* value	AOR (95% CI) *p* value
		Controlled	Uncontrolled		
Sex	Male	44	39	1.323 (0.719–2.434) *p*=0.369	—
	Female	60	43	1.00	—

Age in years	18–39	19	14	1.00	1.00
	40–59	63	38	0.926 (0.393–2.186) *p*=0.861	0.727 (0.232–2.284) *p*=0.585
	≥60	22	30	1.971 (0.785–4.952) *p*=0.149	4.537 (1.142–18.024) *p*=0.032

Marital status	Single	13	8	1.00	—
	Married	73	53	1.163 (0.420–3.219) *p*=0.772	—
	Divorced	12	11	1.607 (0.454–5.688) *p*=0.462	—
	Widowed	6	10	2.500 (0.640–9.766) *p*=0.188	—

Residence	Urban	50	36	1.00	—
	Rural	54	47	1.229 (0.667–2.266) *p*=0.508	—

Educational status	No formal education	46	37	1.178 (0.507–2.739) *p*=0.703	—
	Primary school	24	20	1.299 (0.508–3.319) *p*=0.585	—
	Secondary school	13	11	1.136 (0.376–3.434) *p*=0.821	—
	College/university	21	15	1.00	—

Occupation	Employed	77	66	1.00	—
	Unemployed	27	16	0.698 (0.330–1.477) *p*=0.347	—

Knowledge of hypertension	Good	44	22	1.00	—
	Poor	60	60	1.792 (0.923–3.478) *p*=0.085	—

Duration with hypertension	<2 years	33	24	1.00	1.00
	2–4 years	29	25	1.176 (0.534–2.591) *p*=0.687	2.906 (0.786–10.738) *p*=0.110
	≥5 years	42	33	1.150 (0.552–2.394) *p*=0.209	3.534 (1.062–11.760) *p*=0.040

Cigarette smoking status	Yes	10	15	2.405 (1.025–5.642) *p*=0.044	7.697 (2.356–25.146) *p*=0.001
	No	94	67	1.00	1.00

Alcohol drinking status	Yes	22	19	1.166 (0.565–2.404) *p*=0.678	—
	No	82	63	1.00	—

Presence of comorbidities	Yes	25	25	1.442 (0.737–2.822) *p*=0.225	—
	No	79	57	1.00	—

Adherence	Adherent	45	14	1.00	1.00
	Nonadherent	59	68	3.509 (1.631–7.550) *p*=0.001	6.584 (2.337–18.553) *p* < 0.001

Antidiabetic medications	OHAs alone	60	43	1.00	—
	Insulin alone	40	32	1.180 (0.622–2.238) *p*=0.612	—
	Both OHAs and insulin	4	7	2.662 (0.757–9.368) *p*=0.127	—

Antihypertensive medications	Single AHAs	74	46	1.00	1.00
	Two AHAs	20	26	1.868 (0.925–3.775) *p*=0.082	2.284 (0.818–6.377) *p*=0.115
	Three AHAs	10	10	1.619 (0.609–4.303) *p*=0.334	4.549 (0.960–21.557) *p*=0.056

Glycemic control	Controlled	77	29	1.00	1.00
	Uncontrolled	27	53	8.354 (4.166–16.751) *p* < 0.001	21.630 (8.057–58.070) *p* < 0.001

OHAs = oral hypoglycemic agents; AHAs = antihypertensive agents.

## Data Availability

Data and materials are available from the corresponding author and will be available upon request.

## Abbreviations

ACEI: Angiotensin converting enzyme inhibitors
AHAs: Antihypertensive agents
ARBs: Angiotensin receptor blockers
BP: Blood pressure
CCB: Calcium channel blockers
CVD: Cardiovascular disease
FBG: Fasting blood glucose
HTN: Hypertension
NRH: Nekemte Referral Hospital
OHAs: Oral hypoglycemic agents
USA: United States of America.
